# Optimization of Time-Weighted Average Air Sampling by Solid-Phase Microextraction Fibers Using Finite Element Analysis Software

**DOI:** 10.3390/molecules23112736

**Published:** 2018-10-23

**Authors:** Bulat Kenessov, Jacek A. Koziel, Nassiba Baimatova, Olga P. Demyanenko, Miras Derbissalin

**Affiliations:** 1 Center of Physical Chemical Methods of Research and Analysis, Al-Farabi Kazakh National University, Almaty 050012, Kazakhstan; baimatova@cfhma.kz (N.B.); demyanenko@cfhma.kz (O.P.D.); derbissalin@cfhma.kz (M.D.); 2 Department of Agricultural and Biosystems Engineering, Iowa State University, Ames, IA 50011, USA; koziel@iastate.edu

**Keywords:** solid-phase microextraction, air sampling, air analysis, volatile organic compounds, COMSOL, time-weighted average

## Abstract

Determination of time-weighted average (TWA) concentrations of volatile organic compounds (VOCs) in air using solid-phase microextraction (SPME) is advantageous over other sampling techniques, but is often characterized by insufficient accuracies, particularly at longer sampling times. Experimental investigation of this issue and disclosing the origin of the problem is problematic and often not practically feasible due to high uncertainties. This research is aimed at developing the model of the TWA extraction process and optimization of TWA air sampling by SPME using finite element analysis software (COMSOL Multiphysics, Burlington, MA, USA). It was established that sampling by porous SPME coatings with high affinity to analytes is affected by slow diffusion of analytes inside the coating, an increase of their concentrations in the air near the fiber tip due to equilibration, and eventual lower sampling rate. The increase of a fiber retraction depth (*Z*) resulted in better recoveries. Sampling of studied VOCs using 23 ga Carboxen/polydimethylsiloxane (Car/PDMS) assembly at maximum possible *Z* (40 mm) was proven to provide more accurate results. Alternative sampling configuration based on 78.5 × 0.75 mm internal diameter SPME liner was proven to provide similar accuracy at improved detection limits. Its modification with the decreased internal diameter from the sampling side should provide even better recoveries. The results obtained can be used to develop a more accurate analytical method for determination of TWA concentrations of VOCs in air using SPME. The developed model can be used to simulate sampling of other environments (process gases, water) by retracted SPME fibers.

## 1. Introduction

Analysis of time-weighted average (TWA) concentrations of volatile organic compounds (VOCs) in outdoor and indoor (occupational) air is an important part of environmental monitoring programs aiming at chronic exposure or background concentrations. Such analysis is commonly conducted using gas chromatography (GC) in combination with various sampling and sample preparation approaches. Passive sampling is a common approach for determination of TWA concentrations because of its simplicity and low cost. However, most techniques require additional sample preparation and thermal desorption in a separate unit connected to a GC [1]. 

**Figure 1:**
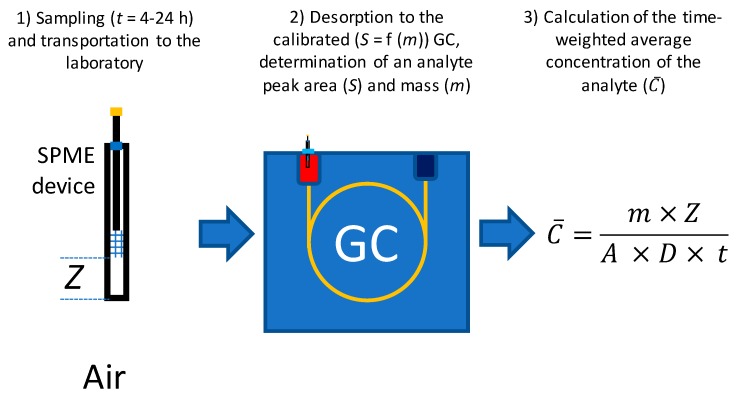
The typical procedure of time-weighted average sampling and analysis using retracted solid-phase microextraction fiber.

Solid-phase microextraction (SPME) is the only TWA sampling technique, that does not require additional stages and/or equipment [2]. It is based on sampling via the passive VOCs extraction by a fiber coating retracted inside a protecting needle followed by thermal desorption inside a GC injection port [3,4]. Desorption of VOCs from the SPME coating is fast and does not require cryogenic or another type of focusing as is the case with whole air- or sorbent tube-based samples [5]. In the TWA mode, the SPME device with retracted fiber is deployed into a sampling location for the desired period (e.g., 24 h for daily average sampling), then isolated from possible interferences during storage and transport to a laboratory and analyzed (Figure 1). The method can be considered “green” because it fulfills all the requirements of green analytical chemistry [6,7].

Calibration is relatively simple compared with a “classic” exposed SPME fiber that is subject to variable thickness of the boundary layer that affects the rate of extraction [8,9]. TWA sampling by retracted SPME fibers is described by the simplified version of the Fick’s law of diffusion [3]:

(1) $$\bar{C}=\frac{n\times Z}{A\times D\times t}$$

where $\bar{C}$—TWA concentration of an analyte, mol·m^−3^; *n*—amount of an analyte extracted by a coating, mol; *Z*—diffusion path length (distance between the needle opening to the tip of the retracted fiber), m; *A*—internal cross-section area of a protecting needle, m^2^; *D*—gas-phase molecular diffusion coefficient for a VOC, m^2^·s^−1^; *t*—sampling time, s.

Equation (1) can also be interpreted by an extraction process, i.e., the amount of analyte extracted is proportional to TWA concentration outside of the SPME needle opening, needle opening area, sampling time, and the gas-phase molecular diffusion coefficient, and inversely proportional to retraction depth.

Several important assumptions are made with the application of Equation (1) to TWA-SPME, i.e., (1) the fiber coating acts as a “zero sink” (without desorption of analytes) and does not affect the rate of sampling; (2) the SPME fiber coating is consistent and reliably responding to changing concentrations in the bulk gas-phase outside of the needle opening; and (3) the gas-phase concentration in the bulk are the same as at the face of the fiber needle opening.

To date, all published research on TWA-SPME has used Equation (1) as the basis of quantification [3,4,10,11,12,13,14,15,16,17,18,19] of VOCs in an outdoor air, laboratory air, pyrolysis reactor air, engine exhaust, and process air. Equation (1) predicted measured gas concentrations with reasonable accuracy and precision. However, more evidence suggests that the discrepancies between the model and experimental data exist. Woolcock et al. [17] reported a significant departure from the zero-sink assumption and from Equation (1) suggesting “apparent” diffusion coefficient (*D*) dependent on both sampling time (*t*) and retraction depth (*Z*). Baimatova et al. [11] reported significant differences in the extracted mass of naphthalene gas for different SPME coatings, i.e., that Equation (1) does not incorporate. Recent research by Tursumbayeva [20] shows that the discrepancy between Equation (1) and experimental data are amplified when a wide-bore glass liner is used for passive sampling with SPME fiber retracted inside it. Work by Tursumbayeva [20] suggests that not only the tip of the fiber coating (at the physical retraction depth *Z*) is involved in extraction, but the whole fiber coating surface with an “apparent” *Z* that is ~55% longer. Apparent saturation sorption kinetics might also be involved as predicted by Semenov et al. (2000) [21]. Thus, research is warranted to address apparent problems with the use of Equation (1).

Despite the simplicity, quantification of TWA concentrations of VOCs in ambient air using SPME can be associated with poor accuracy and precision [19]. Possible problems are variability of extraction efficiencies associated with inherent and acquired variability between individual SPME fibers, adsorption of analytes by metallic surfaces [16,19], effects of sampled air temperature and humidity.

Experimental optimization of the gas sampling process is very time-consuming, particularly at longer extraction times (>24 h). Such experimental setups are quite complex, and difficult to build and properly maintain in steady-state conditions (e.g., without leaks and with minimal impact of sorption onto the system itself). During experiments, the sensitivity of the analytical instrument can change leading to additional uncertainties. Uncertainties during experimental method optimization do not allow studying effects of parameters having potentially minor impacts on accuracy and precision.

Numerical simulation could provide useful data at various sampling parameters in a much faster and more accurate way. It could also allow modeling of the sensitivity of Equation (1) to ranges of practical (user controlled) parameters for air sampling with retracted SPME. COMSOL Multiphysics allowed efficient numerical modeling of the SPME process using a finite element analysis-based model [22,23,24,25,26,27] for liquid-phase extraction and absorption by SPME coating. Using this approach, it was possible to predict sampling profiles of analytes, which were consistent with experimental data.

The goal of this research was to develop a model for SPME with both absorptive and adsorptive fibers located (retracted) inside a protecting needle using a finite element analysis-based model (COMSOL Multiphysics) and use it to disclose potential sources of inaccuracies in the quantification of time-weighted average concentrations of VOCs in ambient air. Specifically, the effects of SPME sampling time, coating type, diffusion coefficient, fiber coating-gas distribution constant, the internal diameter of protecting needle, and SPME retraction depth on extraction were modeled for several common VOCs. Based on the results of the modeling, alternative sampling geometries were proposed.

## 2. Results and Discussion

### 2.1. Time-Weighted Average (TWA) Sampling Profiles of Benzene from Air Using Different Coatings

**Figure 2:**
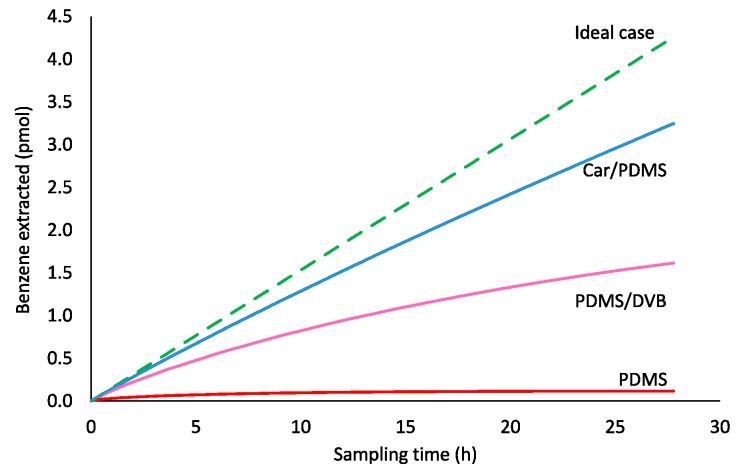
Benzene sampling profiles from ambient air (*T* = 298 K, *Z* = 10 mm, 24 ga needle, *p* = 1 atm, *C_benzene_* = 0.641 μmol·m^−3^) obtained using different fiber coatings. The ideal case pertains to Equation (1).

A sampling of VOCs from the air via retracted SPME has been described using a simplified form of the Fick’s first law of diffusion (Equation (1)). However, this equation works only when a SPME fiber acts as a “zero sink” sorbent. Modeling using COMSOL Multiphysics software (methodology is provided in the Materials and Methods section) allowed obtaining sampling profiles for benzene (Figure 2). Closer inspection of Figure 2 illustrates that none of the studied coatings behave as “zero sink” sorbent adhering to Equation (1), an effect amplified by extended sampling time. After 100,000 s of sampling, Carboxen/polydimethylsiloxane (Car/PDMS), polydimethylsiloxane/divinylbenzene (PDMS/DVB), and polydimethylsiloxane (PDMS) extracted 77, 38 and 2.7%, respectively, of the theoretically required for a passive sampling technique. Even if sampling time is decreased to 10,000 s, recoveries for these three SPME fiber coatings were 91, 69 and 12.6%, respectively. At sampling time 1000 s, recoveries were 97, 88 and 32% for Car/PDMS, PDMS/DVB and PDMS, respectively.

**Figure 3:**
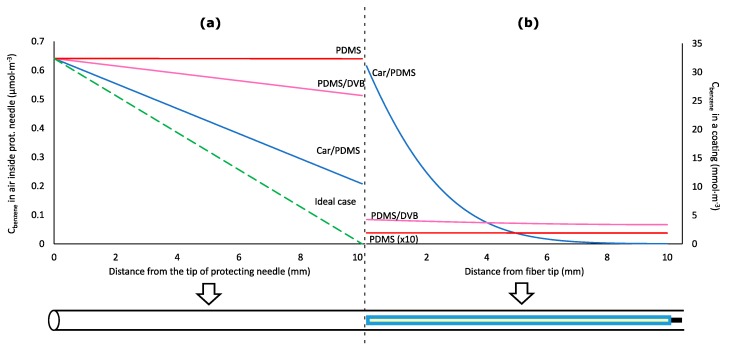
Concentrations of benzene in diffusion path air (**a**) and coating (**b**) of the retracted solid-phase microextraction (SPME) device after 100,000 s of time-weighted average (TWA) air sampling at *Z* = 10 mm.

One possible explanation for the departure from Equation (1) is that it can be caused by the increase of the analyte concentration in the air near the fiber tip (Figure 3a), which is directly proportional to the analyte concentration in the fiber tip continuously increasing during the sampling. The increase of analyte concentration in the air near the fiber tip results in the decrease of the analyte flux (i.e., the number of moles of analyte entering protecting needle per cross-sectional area and time) from the sampled air with time. This affects the sampling rate (i.e., number of moles of an analyte extracted by a coating per unit of time), which was previously assumed to be constant [3,4,10,11,12,13,14,15,16,17,18,19].

SPME fiber coating can affect the apparent rate of sampling. This was previously assumed to be negligible. According to Figure 3, Car/PDMS is the most efficient coating for TWA sampling of benzene because it provides the highest benzene extraction effectiveness indicated by the highest distribution constant. However, sampling by this coating is limited by the slow diffusion of an analyte via pores of the adsorbent (Figure 3b). At sampling time 100,000 s, the closest 1 mm of the Car/PDMS coating to the needle opening contains 41% of the total extracted analyte. Benzene concentration in the fiber tip is about 500 times higher than in its other end (furthest from the needle opening). For PDMS/DVB coating, the concentration in the tip is only about 24% higher. Slower diffusion of benzene via pores of Car/PDMS fiber is caused by the higher affinity of benzene to the surface of the solid phase (higher distribution constant), and lower porosity. Such non-uniform distribution of analytes in the Car/PDMS may be the reason of their slow desorption after TWA sampling and highly tailing peaks, particularly for most volatile analytes, which cannot be cold-trapped and refocused in a column front without cryogens. This problem also decreases the accuracy of the method. 

The accuracy of the model was validated by increasing the pore diffusion coefficient of benzene inside Car/PDMS coating by three orders of magnitude. In this case, the benzene sampling profile was the same as predicted by Equation (1). This also confirms that an analyte diffusion coefficient inside a coating affects sampling profile and the accuracy of its quantification using TWA SPME. The model has also been validated in the 3D mode of COMSOL software, which is much slower compared to 2D (Video S1: COMSOL_TWA_SPME). The difference between the results of 2D and 3D modeling were below 2%, which confirms the accuracy of the 2D model.

### 2.2. Effect of the Diffusion Coefficient and Distribution Constant on Sampling of Analytes by 85-µm Carboxen/Polidimethylsiloxane Coating

**Figure 4:**
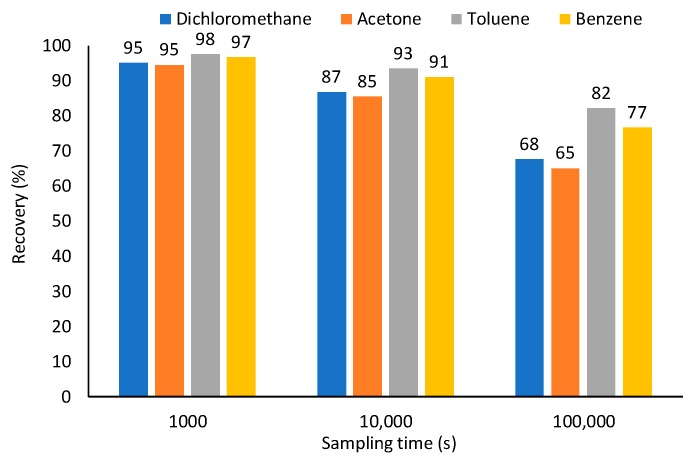
Effect of sampling time of TWA recoveries of analytes having different diffusion coefficients and distribution constants using 85-µm Car/PDMS fiber (*T* = 298 K, *Z* = 10 mm, 24 ga needle, *p* = 1 atm, *C* = 0.641 μmol·m^−3^).

The Car/PDMS coating was used for simulating extraction of other common VOCs associated with a wide range of diffusion coefficients and distribution constants. During 100,000 s, 3.3, 3.9, 3.5 and 3.3 pmol of dichloromethane, acetone, toluene, and benzene, respectively, were extracted, which corresponds to 68, 65, 82 and 77% of the theoretical values predicted by Equation (1) (Figure 4). The lowest value was observed for acetone having a distribution constant close to dichloromethane, and the highest diffusion coefficient among studied compounds. Highest recovery was observed for toluene having the lowest diffusion coefficient and the highest distribution constant. Thus, both diffusion coefficient and distribution constant affect the recovery of sampled analytes. Highest recovery can be achieved at the lowest diffusion coefficient and highest distribution constant. At sampling times 1000 and 10,000 s, recoveries are greater (95–98 and 85–93%, respectively) and less affected by the analyte’s properties.

### 2.3. Effect of a Protecting Needle Gauge Size

**Figure 5:**
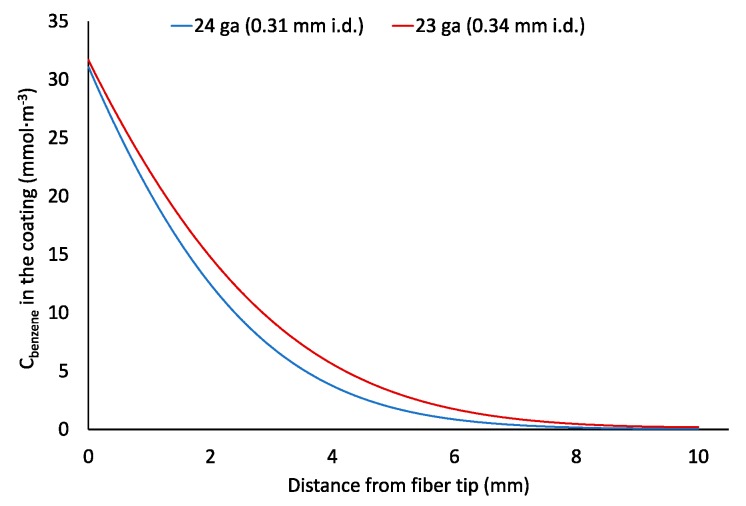
Effect of protecting needle gauge size concentration profile of benzene in the Car/PDMS coating after 100,000 s sampling.

Commercial SPME fiber assemblies are available with two different sizes of a protecting needle 24 ga and 23 ga having an internal diameter (I.D.) 310 and 340 μm, respectively. A cross-section area of the 23 ga needle is 20.3% greater than that of 24 ga needle, which (according to Equation (1)) should result in the proportionally greater amount of an analyte extracted by a 23 ga SPME assembly. However, as shown above, faster extraction rates result in a faster saturation of the coating and lower recovery at longer sampling times. According to the results of COMSOL simulations, despite ~19% greater amounts of extracted analytes compared to a 24 ga assembly, sampling with a 23 ga assembly provided similar recoveries of analytes. Such results can be explained by considering the effect of a greater space between the coating and the internal wall of the protecting needle allowing faster diffusion of analytes to the side and rear sides of the coating (Figure 5). This is consistent with recent experimental observations where straight glass GC liners were used (actual measured I.D. is ~0.84 mm compared with the nominal 0.75 mm I.D.) instead of SPME needle for sampling with retracted fiber [20]. Thus, TWA sampling using 23 ga SPME assembly is recommended over 24 ga for achieving lower detection limits without negative impact on the accuracy. All further modeling was conducted using a 23 ga SPME device.

### 2.4. Effect of Diffusion Path (Z) at Constant Analyte Concentration in Sampled Air

**Figure 6:**
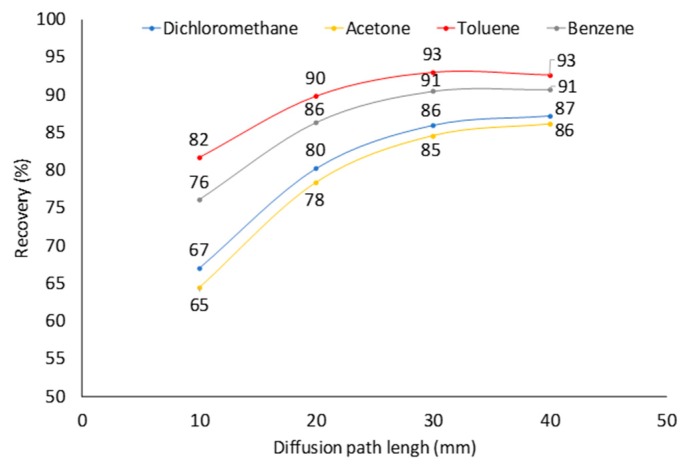
Effect of diffusion path length on recoveries of four analytes (*C* = 0.641 µmol·m^−3^) after sampling for 100,000 s using 23 ga Car/PDMS fiber assembly.

Diffusion path length is one of the two parameters that can easily be adjusted by users for achieving the optimal sampling conditions (the other one being sampling time). The increase of *Z* decreases the rate of sampling. It slows down the saturation of the fiber tip and increases the recoveries of analytes (Figure 6) at longer sampling times. For all studied analytes, at *t* = 100,000 s and *Z* = 40 mm, recovery was 86–93% compared to 66–82% at *Z* = 10 mm (Figure 6). The only major drawback of the increase of *Z* is the decrease of an analyte amount extracted by a coating and a lower analytical signal, which result in the increased detection limits. At *Z* = 40 mm, *C* = 50 µg·m^−3^ (0.641 μmol·m^−3^) and *t* = 100,000 s, 23 ga Car/PDMS assembly extracts ~100 pg of benzene. For GC-mass spectrometry (MS), the detection limit of benzene is less than 2 pg [28] meaning that the detection limit will be ~1 µg·m^−3^, which is five times lower than the maximum permissible annual average concentration of benzene in ambient air in the European Union (5 µg·m^−3^). In other countries, permissible concentrations are even higher.

### 2.5. Effect of Diffusion Path (Z) at Variable Analyte Concentration in Sampled Air (Worst-Case Scenario)

Time-weighted average sampling is conducted during long time periods (e.g., 24 h), during which concentrations of analytes in the sampled air can vary significantly. The apparent worst-case scenario can be when in the first half of sampling, concentration is much higher than during the second half. When the concentration of an analyte in the sampled air becomes close to or lower than the concentration near the fiber tip, the flux of analytes inside a protecting needle can go to a reverse direction resulting in desorption of analytes from a coating. However, this violates the main principle of TWA sampling: the rate of sampling should depend only on the concentration of an analyte in a sampled air. It means that if an analyte concentration in sampled air is zero, a rate of extraction should also be equal to zero. Thus, the aim of this part of the work was to model such a case and estimate the highest possible uncertainty of the TWA SPME sampling approach.

**Figure 7:**
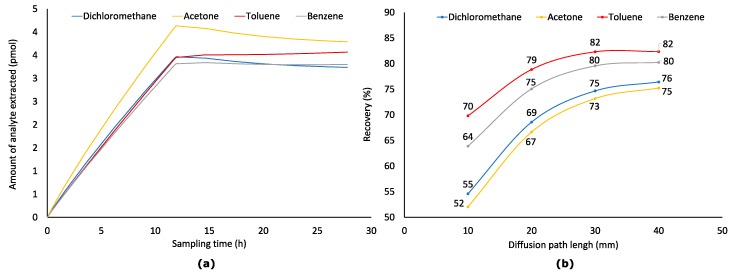
Sampling (*Z* = 10 mm) profiles (**a**) of four analytes from air having their varying concentrations (*C*_0–49,000 s_ = 1.176 μmol·m^−3^, *C*_49,000–51,000 s_ = 1.176–0.1176 μmol·m^−3^, *C*_51,000–100,000 s_ = 0.1176 μmol·m^−3^) and recoveries of analytes (**b**) at *t* = 100,000 s and different *Z*.

As was assumed, desorption of dichloromethane, acetone, and benzene from a fiber started after concentrations of analytes dropped from 1.176 to 0.1176 μmol·m^−3^ in the middle of the extraction process (Figure 7). Desorption of toluene was not observed because it has the highest distribution constant among all studied analytes. However, the toluene sampling rate after the drop of its concentration in sampled air was lower than theoretical. Recoveries of analytes at *Z* = 10 mm dropped from 65–82 to 52–70%, at *Z* = 20 mm from 78–90 to 67–79%, at *Z* = 30 mm from 85–93 to 73–82, at *Z* = 40 mm from 86–93 to 75–82% (Figure 7). Only at *Z* = 40 mm, it was possible to keep recovery of all analytes above 75%. Thus, if possible, for greater accuracy, sampling must be arranged so that no significant drop in concentration takes place. Such a drop can be observed, e.g., if the end of sampling is planned for the night when VOCs concentrations in ambient air are typically lower due to much lower road traffic and other human activities. Also, using shorter sampling times can minimize the risk of the reverse diffusion when ambient concentrations are predicted to drop significantly.

### 2.6. Alternative Geometries for TWA SPME Sampling

As was shown above (Figure 5), an increase of the internal diameter of a protecting needle provides more space for analytes to diffuse around the coating and better reach the side of the coating. It decreases the controlling role of the fiber coating tip and should lead to more accurate and reproducible results.

**Figure 8:**
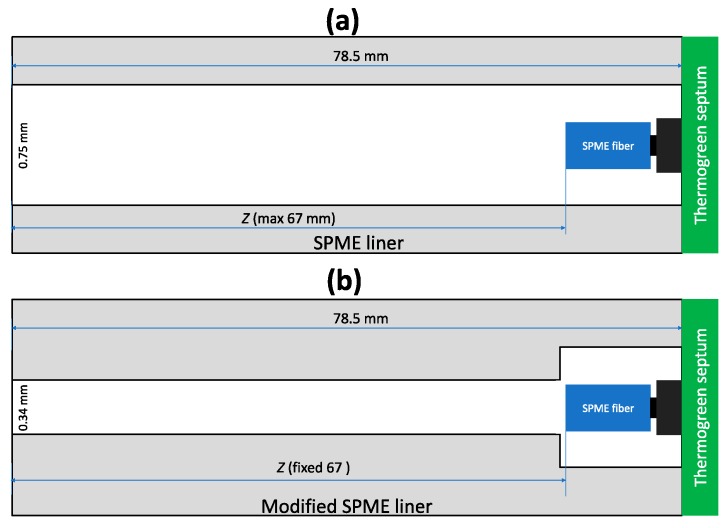
Alternative geometries for TWA SPME sampling: (**a**) used by Tursumbayeva [18], and (**b**) proposed in this research to minimize sources of deviation from Fick’s law of diffusion calibration.

**Figure 9:**
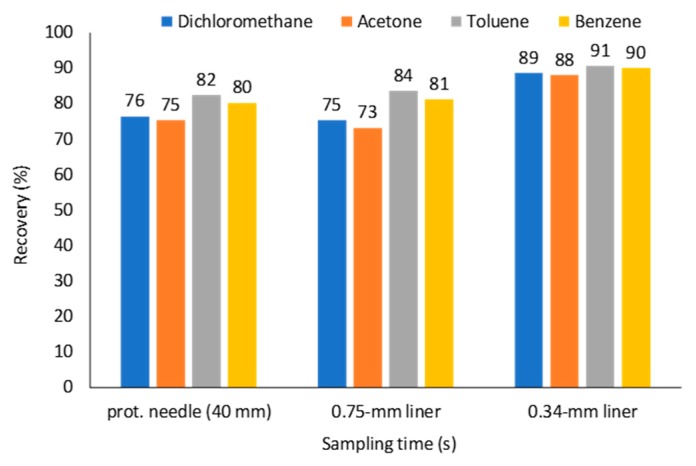
Effect of TWA SPME sampling geometry on recoveries (*t* = 100,000 s, *C*_0–49,000 s_ = 1.176 μmol·m^−3^, *C*_49,000–51,000 s_ = 1.176–0.1176 μmol·m^−3^, *C*_49,000–100,000 s_ = 0.1176 μmol·m^−3^).

Tursumbayeva [20] proposed using SPME liner for TWA SPME to avoid sorption of analytes onto metallic walls of a protecting needle. The same approach can be used to avoid equilibration of analytes between the fiber tip and the surrounding space after sampling over longer time periods. At variable concentrations of analytes (as simulated in the previous section), calculated recoveries for VOCs using *Z* = 67 mm (Figure 8a) are 73–84%, which are close to the values obtained using retracted fiber at *Z* = 40 mm. No improvement was observed because of 0.75-mm I.D. SPME liner has 4.9 times greater cross-sectional area than 23 ga protecting needle, which results in 2.9 times greater theoretical flux of analytes from sampled air to the coating under the set *Z* (67 and 40 mm, respectively). To decrease the flux of analytes, the liner can be manufactured with a lower I.D. (e.g., 0.34 mm as for 23 ga needle) from the sampling side almost to the expected location of the fiber as shown in Figure 8b. Under these conditions, recoveries increased to 88–91% (Figure 9).

**Figure 10:**
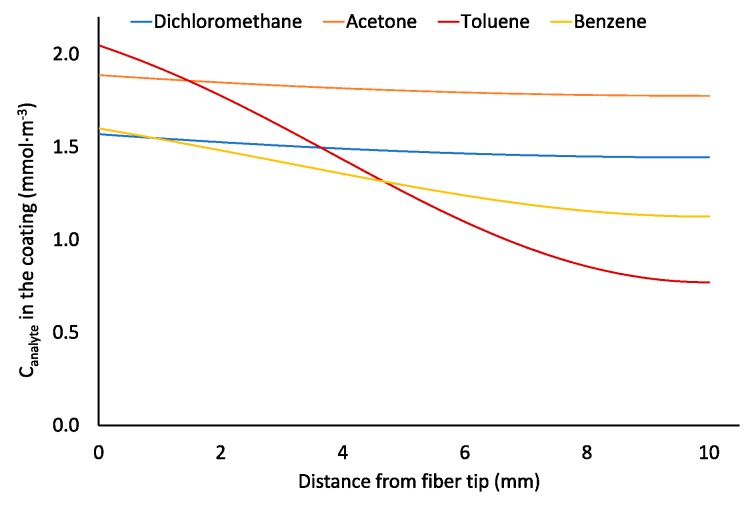
Profiles of analyte concentration in the Car/PDMS coating after sampling ambient air (*C*_0–49,000 s_ = 1.176 μmol·m^−3^, *C*_49,000–51,000 s_ = 1.176–0.1176 μmol·m^−3^, *C*_49,000–100,000 s_ = 0.1176 μmol·m^−3^) for 100,000 s using the geometry presented in Figure 8a.

The use of alternative geometries (Figure 10) resulted in a more uniform distribution of the analytes in the coating; for 0.75-mm I.D. SPME liner concentrations of analytes near the fiber tip were only 1.1–2.7 times greater than at another side of the coating. This should result in faster desorption of analytes, less pronounced peak tailing and greater accuracy of the method. A similar effect is achieved when using Radiello^®^ passive air sampler [29], which provide a greater surface area of an adsorbent available for the diffusive air sampling.

## 3. Materials and Methods

### 3.1. General Parameters of Modeling

Simulations were completed using COMSOL Multiphysics 5.3a (Burlington, MA, USA) on a desktop computer equipped with quad-core Core i5 processor and 8 Gb of random-access memory. For modeling, “Chemical Species Transport” module (“Transport of diluted species” or “Transport of diluted species in porous media” physics) was used in “Time-Dependent” mode in two dimensions (axisymmetric). Fick’s second law of diffusion was used by the module:

(2) $$\frac{\partial c_{i}}{\partial t}=\nabla \times \left(D_{i}\times \nabla c_{i}\right)$$

Benzene, a ubiquitous air pollutant, was used as a model analyte for most initial calculations. Diffusion coefficients of benzene in the air and PDMS coating were set to 8.8 × 10^−6^ and 10^−10^ m^2^·s^−1^, respectively [30]. Distribution constant (*K_d_*) for benzene and common SPME coatings was set to 150,000 (85 µm Car/PDMS) [31], 8300 (65 µm PDMS/DVB) [31], and 301 (PDMS) [5]. For dichloromethane, acetone and toluene, distribution constants between 85 µm Car/PDMS coating and air were set to 72,000, 71,000 and 288,000, respectively [31].

The geometry of a fiber assembly was built in as inputs based on the data provided by Pawliszyn [5]. Simulations were conducted for Stableflex^®^ (Supelco, Bellefonte, PA, USA) fibers with a core diameter of 130 µm. For 85 µm Car/PDMS and 65 µm PDMS/DVB, total fiber diameters were set to 290 and 270 µm, respectively. Calculations were conducted for 24- and 23 ga coatings having internal diameter of 310 and 340 µm, respectively.

The extra fine free triangular mesh was used for the modeling. To provide better meshing at the coating−air interface, the resolution of narrow regions was increased to “2”. The computation was completed in the range between 0 and 100,000 s at the step of 1000 s. The concentration of an analyte at the tip of the protecting needle was set to 0.641 µmol·m^−3^, which corresponds to 50 µg·m^−3^ of benzene.

### 3.2. Sampling Using Absorptive Coatings

**Figure 11:**
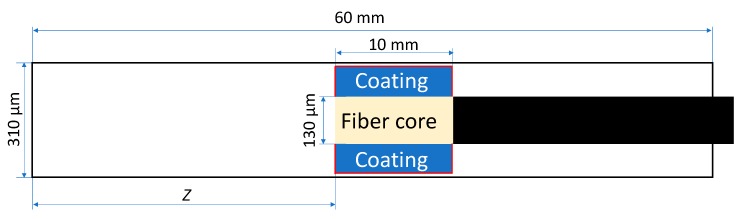
The geometry of SPME device (retracted inside a protective needle for TWA sampling) used for modeling. Note: red lines indicate the boundaries between air and coating.

Inward (and outward) fluxes from (or backward to) air into an absorptive coating (*Flux*_1_ and *Flux*_2_, respectively) at the boundaries (marked by red lines in Figure 11) were simulated using the equation, previously proposed by Mackay and Leinonen [32] for the water−air interface:

(3) $$Flux_{1}=k\times \left(C_{a}-\frac{C_{f}}{K_{d}}\right);\;Flux_{2}=k\times \left(\frac{C_{f}}{K_{d}}-C_{a}\right)$$

where: *k*—flux coefficient, m·s^−1^; *C_a_* and *C_f_*—concentrations of an analyte in air and coating at the boundary layer, respectively, mol·m^−3^; *K_d_*—distribution constant for a VOC between SPME coating and air.

The true value of the flux coefficient was unknown, but in this research, it was assumed to be sufficiently high for not affecting the flux, as was recently proposed by Alam et al. [23]. Thus, the flux coefficient was set to 1000 m·s^−1^. A further increase of the flux coefficient did not affect the results of the modeling.

### 3.3. Sampling Using Adsorptive Coatings

For adsorptive coatings, the “Adsorption” mechanism was activated in the model. The isotropic diffusion coefficient (in the air inside pores) was the same as for air (set to 8.8, 8.7, 12.4 and 10.1 mm^2^·s^−1^ for benzene, toluene, acetone, and dichloromethane, respectively). The approach proposed by Mocho and Desauziers [33] involving Knudsen diffusion in micro-pores was also tested. However, it was later rejected for model simplification because the diffusion of analytes inside coating is mainly driven by molecular diffusion inside macro-pores. The presence of PDMS binder was not considered in the model because: (1) it has much weaker affinity to analytes than Carboxen; and (2) the layer of PDMS in the coating is very thin and should not affect the diffusion of analytes [5]; (3) there is not enough published information about the exact structure of the coating.

Adsorption was set to “User defined” with a distribution constant (*K_p_*, m^3^·kg^−1^) calculated as a dimensionless distribution constant divided by a coating density (*K_d_*/*ρ*). Coating porosities (*ε* = 0.685 for Car/PDMS and 0.775 for PDMS/DVB) were calculated using intra-particle porosities (0.37 for Car, and 0.55 for DVB [34]) and inter-particle porosity. The exact value of the latter is proprietary and not available in the open literature. Taking into account, the spherical shape of particles and available scanning electron microscope (SEM) photos, the inter-particle porosity of both coatings was set to the maximum possible value (0.50). A particle porosity (*ε*) was calculated as the total volume of pores (0.78 mL for Car, and 1.54 mL for DVB) divided by the total volume of one gram of material (2.13 mL for Car, and 2.78 mL for DVB). Densities of the coatings were calculated using free fall densities of the particles (470 kg·m^−3^ for Car, and 360 kg·m^−3^ for DVB) [34] and inter-particle porosity. Effective diffusion coefficients were calculated during the calculations by the COMSOL software using the Tortuosity model [33]:

(4) $$D_{e}=\frac{\varepsilon \times D_{p}}{\tau }$$

where: *ε*—porosity; *τ*—tortuosity calculated from the porosity [33]:

(5) τ=ε+1.5×(1−ε)

For Car/PDMS and PDMS/DVB coatings, tortuosity was set to 1.16 and 1.1125, respectively.

## 4. Conclusions

A finite element analysis-based model (based on COMSOL Multiphysics software) allowed efficient simulation of TWA air sampling of VOCs using retracted SPME fibers. It was possible to model the effects of sampling time, coating type (including adsorptive coatings for the first time) and composition, diffusion coefficient, the distribution constant, the internal diameter of a protecting needle and diffusion path on the recovery of analytes, their concentration profiles in the air inside protecting needle, and the coating. The advantages of such a simulation compared to an experiment are: (1) time and cost savings; (2) lower uncertainty and the possibility to discover minor impacts of sampling parameters on its performance; and (3) the possibility to understand and optimize a sampling process in greater detail. The results of this research allowed disclosing potential sources of the apparent departure from Fick’s law of a diffusion-based model used for quantification of VOCs with retracted SPME.

It was established that sampling by porous coatings with high affinity to the analyte (Car/PDMS) is affected by the saturation of the fiber tip and slow diffusion of analytes in the coating. Highest recoveries are achieved for analytes having lowest diffusion coefficients and highest affinities to a coating. The increase of an internal diameter of a protecting needle from 24 to 23 ga allows proportionally greater responses to be obtained at similar recoveries.

The most important parameter of a sampling process that users can control is a retraction depth. The increase of *Z* allows slowing down the sampling and achieving higher recoveries of analytes. In this study, at *Z* = 40 mm and constant analyte concentration in a sampled air, recoveries of studied analytes reached 86–93% compared to 65–82% at *Z* = 10 mm. The developed model allowed simulation of the worst sampling case when analyte concentrations significantly drop in the middle of sampling. For the first time, it has been proven that at such sampling conditions and *Z* = 40 mm, recoveries of analytes can drop by ~10%, while at *Z* = 10 mm by ~15%.

According to the results of the simulation, it is optimal to conduct sampling of studied VOCs using a 23 ga Car/PDMS assembly at *Z* = 40 mm. Expected detection limits at these parameters are about 1 µg·m^−3^.

Alternative geometries of a protective TWA SPME sampling devices could be used to increase recoveries of analytes. Sampling using 0.75-mm I.D. SPME GC liner at *Z* = 67 mm provides similar recoveries compared to sampling using a protecting needle at *Z* = 40 mm, but it provides greater amounts of analytes extracted and lower detection limits. To achieve greater recovery, part of the liner should have narrower I.D. (e.g., 0.34 mm). The increase of the diameter of the extraction zone where the coating is located results in a more uniform distribution of analytes, which should lead to faster desorption, less pronounced peak tailing and greater accuracy. Specific sampler parameters should be selected for particular sampling time and environmental conditions (temperature and atmospheric pressure) using the developed model.

The methodology used in this study could also be used for more accurate and simpler calibration of the method. It can be used to model the sampling of other environments (process gases, water) by retracted SPME fibers. Further modification of this model could allow simulation of soil and soil gas sampling.

## Supplementary Materials

The following are available online at http://www.mdpi.com/1420-3049/23/11/2736/s1. Video S1: COMSOL_TWA_SPME.
